# RNA sequencing analysis of altered expression of long noncoding RNAs associated with *Schistosoma japonicum* infection in the murine liver and spleen

**DOI:** 10.1186/s13071-020-04457-9

**Published:** 2020-12-01

**Authors:** Tianqi Xia, Bikash Ranjan Giri, Jingyi Liu, Pengfei Du, Xue Li, Xuxin Li, Shun Li, Guofeng Cheng

**Affiliations:** 1 Shanghai Veterinary Research Institute, Chinese Academy of Agricultural Sciences, Key Laboratory of Animal Parasitology, Ministry of Agriculture and Rural Affairs, Shanghai, 200241 People’s Republic of China; 2 Shanghai Tenth People’s Hospital, Institute for Infectious Diseases and Vaccine Development, Tongji University School of Medicine, 301 Middle Yanchang Road, Shanghai, 200072 People’s Republic of China; 3 Tongji University School of Medicine, 1239 Si-ping Road, Shanghai, 200092 People’s Republic of China

**Keywords:** *Schistosoma japonicum*, Long noncoding RNA, RNA sequencing, Liver, Spleen, Pathogenesis

## Abstract

**Background:**

Schistosomiasis is a chronic, debilitating infectious disease caused by members of the genus *Schistosoma*. Previous findings have suggested a relationship between infection with *Schistosoma* spp. and alterations in the liver and spleen of infected animals. Recent reports have shown the regulatory role of noncoding RNAs, such as long noncoding RNAs (lncRNAs), in different biological processes. However, little is known about the role of lncRNAs in the mouse liver and spleen during *Schistosoma japonicum* infection.

**Methods:**

In this study, we identified and investigated lncRNAs using standard RNA sequencing (RNA-Seq). The biological functions of the altered expression of lncRNAs and their target genes were predicted using bioinformatics. Ten dysregulated lncRNAs were selected randomly and validated in reverse transcription-quantitative real-time polymerase chain reaction (RT-qPCR) experiments.

**Results:**

Our study identified 29,845 and 33,788 lncRNAs from the liver and spleen, respectively, of which 212 were novel lncRNAs. We observed that 759 and 789 of the lncRNAs were differentially expressed in the respective organs. The RT-qPCR results correlated well with the sequencing data. In the liver, 657 differentially expressed lncRNAs were predicted to target 2548 protein-coding genes, whereas in the spleen 660 differentially expressed lncRNAs were predicted to target 2673 protein-coding genes. Moreover, functional annotation showed that the target genes of the differentially expressed lncRNAs were associated with cellular processes, metabolic processes, and binding, and were significantly enriched in metabolic pathways, the cell cycle, ubiquitin-mediated proteolysis, and pathways in cancer.

**Conclusions:**

Our study showed that numerous lncRNAs were differentially expressed in *S. japonicum*-infected liver and spleen compared to control liver and spleen; this suggested that lncRNAs may be involved in pathogenesis in the liver and spleen during *S. japonicum* infection.
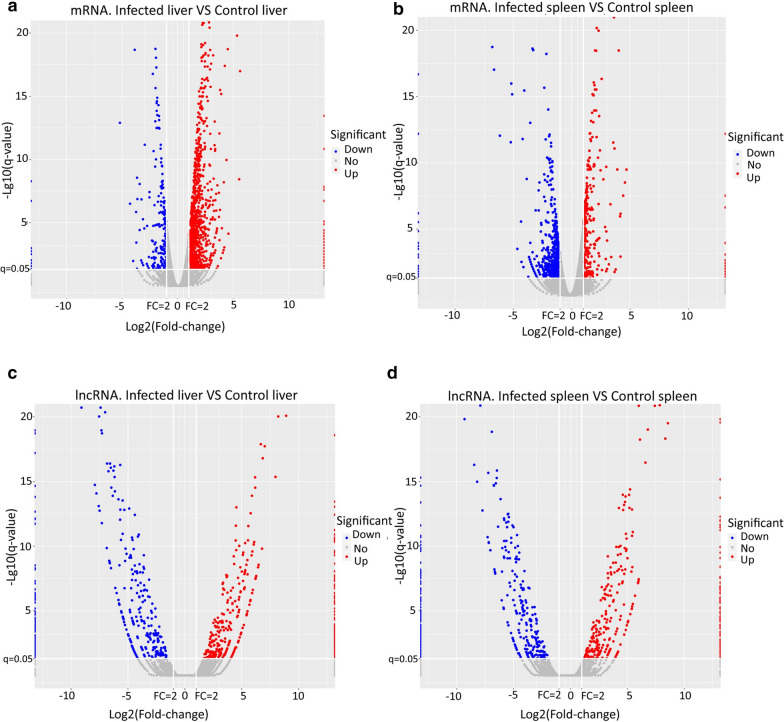

## Background

Schistosomiasis is a devastating acute and chronic disease that afflicts over 200 million people in 76 tropical and subtropical countries [1]. It contributes to approximately 200,000 deaths annually and is considered a major health hazard in Africa, the Middle East, and Southeast Asia, despite enormous control measures [2]. Upon infection, schistosome cercariae develop into adults in the final host portal-mesenteric venous system. Most of the eggs produced by mature females are mainly lodged in the liver, intestine and other tissues, and result in tissue and organ damage [3]. Schistosomiasis is also described by extensive splenomegaly, which is considered a cause of morbidity and mortality in this disease [4]. Although extensive studies have been conducted to dissect the molecular mechanisms of hepatic pathology, the immunopathology of schistosome-induced splenomegaly has been largely neglected. Previously, *Schistosoma* infection was shown to induce significant splenomegaly characterized by a loss of definition between the red and white pulp, as well as altered immune responses to other infections [5, 6]. The precise molecular mechanisms and transcriptional modulation corresponding to these cellular and immunological changes, however, have not been fully demonstrated.

Noncoding RNAs (ncRNAs) are generally divided into two classes based on the sequence length cutoff. RNA molecules that are under 200 nucleotides long are referred to as small ncRNAs; this class includes microRNAs (miRNAs) and endogenous small interfering RNAs. RNA molecules that have more than 200 bases are known as long noncoding RNAs (lncRNAs) [7]. Previously, lncRNAs were considered to be nonfunctional; however, recent studies have revealed their roles in numerous biological processes (including development, differentiation, cancer progression, and cell metabolism) at both the transcriptional and post-transcriptional levels [8–10]. Recently, several reports have demonstrated that lncRNAs are induced and modulate both innate and adaptive immune responses [11, 12]. lncRNAs regulate all processes involved in RNA metabolism including chromatin modification, transcription, splicing, RNA transport, and translation [13]. lncRNAs are transcribed from intergenic and intronic regions, as well as from overlapping regions [13, 14], and mechanistically provide platforms for assembling RNA–protein-complex guides that recruit RNA–protein complexes to target genes and sequester regulatory proteins away from their target DNA sequences [15, 16]. Based on the genomic region of origin and nearby protein-coding genes, lncRNAs can be classified into several categories including long-intergenic noncoding RNAs, natural antisense RNAs, overlapping RNAs, bidirectional RNAs, and sense intronic RNAs [17]. Dysregulation of lncRNAs has been associated with several human cancers and other diseases [18]; however, the cellular factors that regulate their expression have not been clearly identified. The involvement of specific lncRNAs in hepatic diseases has been described, and hepatic stellate cells trans-differentiation is regulated by different lncRNAs [19–21]

A previous study employed different tools to study differential gene expression patterns occurring in response to *Schistosoma* infections [22]. Recently, *Schistosoma* lncRNAs have also been investigated for their potential functions [23–29]. However, lncRNAs in *Schistosoma japonicum*-infected mouse liver and spleen samples have not been investigated by RNA-Seq. In this study, we identified and analyzed the lncRNA expression profiles in the liver and spleen of control and *Schistosoma*-infected animals at 25 days after infection began. A total of 212 novel lncRNAs were identified and 1548 lncRNAs were differentially expressed in the liver and spleen, after* S. japonicum* infection. We also annotated the possible functions of the predicted lncRNAs via Gene Ontology (GO) and Kyoto Encyclopedia of Genes and Genomes (KEGG) analysis, which showed that they are involved in binding in cellular processes, metabolic pathways, the cell cycle, and ubiquitin-mediated proteolysis. Several lncRNAs with altered expression were selected for validation by reverse transcription-quantitative real-time polymerase chain reaction (RT-qPCR). Our results are expected to contribute to an understanding of the roles of lncRNAs during *S. japonicum* infection in the liver and spleen.

## Methods

### Animal experiments

All animal experiments were carried out in strict accordance with the recommendations in the Guide for the Care and Use of Laboratory Animals of the Ministry of Science and Technology of the People’s Republic of China. All animal care and experimental procedures were approved by the Animal Management Committee and the Animal Care and Use Committee of Shanghai Science and Technology Commission of Shanghai municipal government for the Shanghai Veterinary Research Institute, Chinese Academy of Agricultural Sciences, People’s Republic of China (permit no. SYXK 2016–0010).

Twenty healthy, pathogen-free male BALB/c mice (aged 6 weeks) were obtained from Shanghai Jiesijie Experimental Animal (Shanghai). The mice were randomly divided into (1) a control group (uninfected group), and (2) a *S. japonicum*-infected group (ten mice per group). Mice in the latter group were percutaneously infected with approximately 120 *S. japonicum* cercariae (Anhui isolate; People’s Republic of China). At 25 days post-infection, the mice were euthanized, and liver and spleen tissues were collected aseptically and stored in liquid nitrogen until needed.

### RNA isolation, library construction, and Illumina sequencing

Total RNA was isolated from liver and spleen tissues using TRIzol (Invitrogen, Life Technologies, USA) following the manufacturer’s protocol. The RNA purity and integrity were checked using a NanoDrop ND-1000 (Thermo Fisher Scientific, Carlsbad, CA) and an Agilent Bioanalyzer 2100 (Agilent Technologies, Santa Clara, CA). Quantified total RNA was further purified using a RNeasy Micro Kit (Qiagen, Hilden, Germany), and then the ribosomal RNA was depleted using a Ribo-Zero rRNA Removal Kit (Epicentre, CA), following the manufacturer’s protocol. Following purification, the RNA was fragmented into small pieces, the first and second complementary DNA (cDNA) strands were synthesized, poly-A 3′ ends were added, adapters were ligated, and the fragments were enriched to construct the RNA-Seq library according to the Illumina TruSeq RNA sample-preparation guide. The library quality was assessed on a Qubit 2.0 Fluorometer (Thermo Fisher Scientific) and an Agilent 2100 Bioanalyzer (Agilent Technologies). Then, four pooled RNA samples (separate liver and spleen RNA samples from infected and uninfected mice; ten RNA samples for each pool) were subjected to 2 × 125 base pair (bp) paired-end sequencing using an Illumina HiSeq 2500 instrument.

### Sequencing data analysis and prediction of lncRNAs

Clean, high-quality data were obtained by processing the raw data using Seqtk (https://www.github.com/lh3/seqtk). Then the clean reads were mapped to the reference genome (mm10) using HISAT software (version 2.0.4) [30]. The mapped transcripts for each sample were independently assembled with Stringtie software (version 1.3.0) [31]. To identify novel lncRNA transcripts, we first compared the assembled transcripts with reference annotations using GffCompare (version 0.9.8) and obtained novel transcripts that failed to match the known annotations. Then, only transcripts class-coded as “i” (a transfrag falling entirely within a reference intron), “u” (unknown, intergenic transcript), and “x” (exonic overlap with a reference sequence on the opposite strand) were selected as candidate lncRNAs; transcripts exceeding 200 nucleotides spanning at least two exons with a predicted ORF of < 300 bp were retained for further analysis. Next, the Coding Potential Calculator (CPC) [32], Coding-Non-Coding Index (CNCI) [33] and Pfam [34] tools were utilized to predict the coding ability of each transcript. The final set of predicted lncRNAs comprised all transcripts with a CPC score < 0, a CNCI score < 0, and a Pfam determined to be not significant. Novel lncRNAs were identified by removing sequences that matched known lncRNAs. These lncRNAs were divided into several categories in terms of the relative proximity to their protein-coding genes, as described previously [17].

### Analysis of differentially expressed lncRNAs and mRNAs

Transcript expression levels were determined as transcripts per million (TPM) [35, 36]. We used edgeR [37] to screen for genes and lncRNAs that were differentially expressed between the infected and control mouse groups, both in the liver and spleen. The mRNAs and lncRNAs showing significant upregulation (fold change ≥ 2 and *q*-value ≤ 0.05) or downregulation (fold change ≤ − 2 and *q*-value ≤ 0.05) were considered to be differentially expressed.

### Predicting the target genes of differentially expressed lncRNAs

For a more comprehensive understanding of the differentially expressed lncRNAs, their targets were predicted. lncRNAs can regulate the expression levels of target protein-encoding genes in* trans* and in* cis* regulation.* Trans*-regulated targets can be predicted based on mRNA sequence complementarity and RNA duplex energy assessments. First, BLAST analysis was performed to select similar or complementary sequences, after which RNAplex [38] was used to calculate the complementary energy between both sequences, and sequences above the threshold (*e* ≤ − 30) were selected as candidate targets. Likewise, the genes transcribed within a 10-kbp window upstream or downstream of the lncRNAs were considered as putative* cis*-regulated target genes.

### GO and KEGG enrichment analysis

To better understand the functions of differentially expressed lncRNAs, we analyzed the functions of differentially expressed lncRNA targets by GO analysis (https://www.geneontology.org) [39]. Briefly, GO analysis classifies genes into hierarchical categories and uncovers associated gene-regulatory networks, based on biological processes and molecular functions. The KEGG analysis is used to understand the high level function and utilities of biological system at the cell, organism and ecosystem level based on molecular-level information generated by different sequencing techniques [40]. Enriched pathways associated with the putative targets of differentially expressed lncRNAs were identified using KEGG (https://www.genome.jp/kegg/). All genes from *Mus musculus* were selected as background genes. Fisher’s exact test was used to calculate* p*-values according to the annotations, and the rich factor was calculated based on the numbers of target genes in the different pathways of interest.

### Prediction of a lncRNA–mRNA network

To further assess the potential interactions and correlations between lncRNAs and mRNAs in the liver and spleen of mice infected with *S. japonicum*, we constructed an lncRNA–mRNA co-expression network using Cytoscape software (version 3.7.2) [41], based on lncRNAs and their* trans*-target mRNAs that were dysregulated.

### RT-qPCR-based validation of lncRNAs and their putative targets

RT-qPCR was performed to validate our RNA-Seq results showing that ten lncRNAs and selected putative targets were differentially expressed in the liver and spleen. Total RNA was isolated from liver and spleen tissues from control and infected mice, using TRIzol (Invitrogen) according to the manufacturer’s protocol. cDNA was reverse-transcribed with the PrimeScript RT Reagent Kit (Takara, Dalian, People’s Republic of China). Then, qPCR was performed in an Eppendorf MasterCycler RealPlex4 instrument (Eppendorf Corporate) using TB Green Premix Ex Taq II (Takara), and glyceraldehyde-3-phosphate dehydrogenase (GAPDH) mRNA expression was detected as an internal control. PCR was performed with an initial denaturation step of 95 °C for 30 s, followed by 40 cycles of 95 °C for 5 s and 60 °C for 30 s. We also evaluated the expression of some putative targets for lncRNAs including transforming growth factor beta 1 (TGFβ-1), Janus kinase 3 (JAK3), and signal transducer and activator of transcription 1 (STAT1) in the liver of mice at 64 days post-infection using RT-qPCR. The relative lncRNA expression levels were calculated following a method described previously [42]. All primers used in this study are listed in Table 1.

**Table 1:** Primers used for reverse transcription-quantitative real-time polymerase chain reaction analysis of long noncoding RNA (*lncRNA*)/gene expression

lncRNA/gene	Forward primer sequences (5′–3′)		Reverse primer sequences (5′–3′)
NONMMUT057813.2	TTAAATGCTGGGCCTGGGTT		ACAGCCTTCTGCACAGTACC
NONMMUT033668.2	CGGGGGAGCATTACCCTTTT		GAGCCAAGCTCCACAGACTT
NONMMUT055723.2	CGTAGAACGCATGGAGTGGA		CCAGGTGGCAACAGGAATCT
NONMMUT050583.2	GGCAAGCAGAATAGGTGGGT		GCCACACCACTTCCTAACCA
NONMMUT036430.2	GCCACTGGGTGCTACACATA		AGAGGCTCACAAGCACAGAC
NONMMUT001686.2	TGTGAAGTGCTGTGGCTAGG		ACTTGGTTCCCCGAGTTGTC
NONMMUT060075.2	CCCAGGGTGATCTTGTTGCT		GCTGGGTCTCCTGATTTGCT
NONMMUT063222.2	AACTGGCCTTTCTCTGCTGG		AGGACTTGACCACCCAGTCT
NONMMUT028556.2	TGGCATTGTTCATGGGGAGT		ACAGACCCTGGGGTGTATTT
NONMMUT033611.2	TGGCATAGAGGCCGTCTAACT		GAAATGAGGCTCCTAGATGTTTAGC
NONMMUT014792.2	TGTCATCACTAACATGGGCCAG		TCATATCGTCATTGAAAGCACAGCC
NONMMUT031907.2	GAAAACTGCTAAGCGCGGTG		GGTCTCAACCATGCAACCCT
NONMMUT061096.2	ACCCCAATTCCTGAGACGTG		GTTCAGAACTTGGCGCCTTC
NONMMUT047510.2	AGGTGTTTTCACCAGCCACA		AGCGAAATGCAAGACGAGGA
ENSMUST00000185516	AGGATTTGTGTCTTGCGTTGTAT		TTCAGTTGCAGGTGTCGGAA
*XCR1*	CACCGAACAGTCAGGCTCAT		GTCCAGTTGCTGAAGGCTCT
*VCAM1*	GAATGAGGGGGCCAAATCCA		GACAGGTCTCCCATGCACAA
*SAMHD1*	CGAGCACTTGCCGAAAAACA		CTCAATGGAGCCCTGTTCGT
*CCR5*	ACACCCTGTTTCGCTGTAGG		GACAGGGTTTAGGCAGCAGT
*GLCCI1*	CCCAACCGGATCAGCTTTCT		GCTGCTCTACGGTCAGTGTT
*IRS1*	AGAACGAGAAGAAGTGGCGG		CCTTTGCCCGATTATGCAGC
*EXO1*	ATGCACACACCCCGTCTAAG		ATTTAGAGGCTGGCCGCTTT
*FRY*	TGTGATGCAGCCAGTTACCTT		GTCTCTGCATCTACGAACGGTG
*CCDC137*	GGACAGCGCAGCAAAGAAAA		CGTAGGCCACATCAGACTCC
*TTYH1*	CTCCTGGTGCTTGTCCTGAG		GTTGGAAGGGGTTGGAGACC
*TGFβ-1*	TGTGTTGGTTGTAGAGGGCAAGGA		TTTGGAGCCTGGACACACAGTACA
*JAK3*	GAGCCAAGTATCCTACCCGC		GAGGAAGGCCTTTGTCCTCC
*STAT1*	AAGTCTGGCAGCTGAGTTCC		TACCACAGGATAGACGCCCA
*GAPDH*	GGTGAAGGTCGGTGTGAACG		ACCATGTAGTTGAGGTCAATGAAGG

* XCR1* Chemokine C motif receptor 1,* VCAM1* vascular cell adhesion molecule 1,* SAMHD1* SAM domain and HD domain 1,* CCR5* chemokine C–C motif receptor 5,* GLCC1* glucocorticoid-induced transcript 1,* IRS1* insulin receptor substrate 1,* EXO1* exonuclease 1,* FRY* furry homolog* Drosophila*,* CCDC137* coiled coil domain containing 137,* TTYH1* tweety homolog 1 *Drosophila, TGFβ-1 transforming growth factor beta 1, JAK3 Janus kinase 3, STAT 1 signal transducer and activator of transcription 1, GAPDH glyceraldehyde-3-phosphate dehydrogenase*

### Statistical analysis

SPSS 21.0 (IBM, Chicago, IL) was used for statistical analysis. The RT-qPCR results are presented as the mean ± SEM, and *p* ≤ 0.05 was considered to reflect a statistically significant difference.

## Results

### Read mapping and transcript assembly

We sequenced control liver, control spleen, infected liver, and infected spleen samples and obtained 84,813,366, 88,151,892, 75,028,306, and 66,281,510 raw reads, respectively. The clean read ratios were 95, 94.8, 98, and 97.5% in the control liver, control spleen, infected liver and infected spleen samples, respectively. After cleaning, trimming and removing the rRNA sequences, we retained 77,044,506, 80,081,036, 72,743,784, and 63,888,244 reads for the control liver, control spleen, infected liver and infected spleen samples, respectively. Using the splice-mapping algorithm of Hisat2 (version 2.0.4), we performed genome mapping with the pre-processed reads. The mapping ratios were 90.6, 94.4, 92.7, and 94% for the control liver, control spleen, infected liver, and infected spleen samples, respectively, when they were mapped to the reference genome.

### Identification and characterization of lncRNAs in murine liver and spleen

**Fig. 1a–d:**
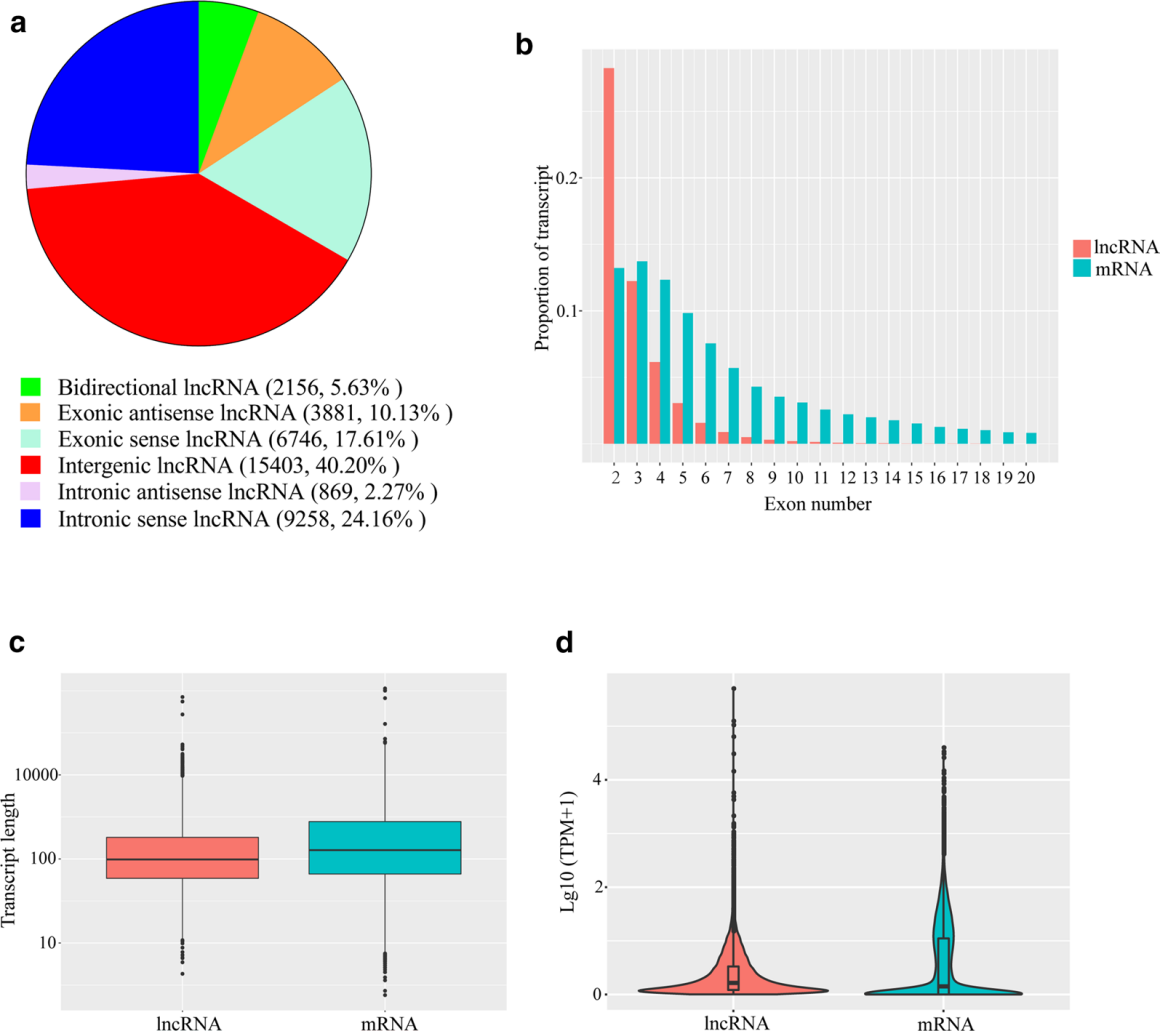
Classification and characteristics of long noncoding RNAs (*lncRNA*s) identified in infected and control murine livers and spleens. **a** Annotations of lncRNAs identified in infected and control liver and spleen specimens. **b** Number of exons per transcript for mRNAs and lncRNAs in livers and spleens of mice. **c** Box plot showing the transcript size distributions for mRNAs and lncRNAs in livers and spleens of mice. **d** Violin plot for lncRNA and mRNAs expression in livers and spleens of mice.* TPM* Transcripts per million

Our bioinformatic analysis identified 34,745 mRNA transcripts and 38,313 lncRNA transcripts (including 212 novel lncRNA transcripts) in the liver and spleen samples. The novel lncRNA transcripts predicted here are listed in Additional file 1: Table S1 and the related mRNAs and lncRNAs are listed in Additional file 2: Tables S2 and Additional file 3: Table S3. Among these lncRNAs, 2156 bidirectional lncRNAs (5.63%), 3881 exonic antisense lncRNAs (10.13%), 6746 exonic sense lncRNAs (17.61%), 15,403 intergenic lncRNAs (40.20%), 869 intronic antisense lncRNAs (2.27%), and 9258 intronic sense lncRNAs (24.16%) were obtained (Fig. 1a; Additional file 4: Table S4). In addition, we characterized the basic genomic features of lncRNAs and compared these features with mRNAs expressed in the liver and spleen. The lncRNA structures were compared with those of the mRNAs, and differences in transcript lengths, exon numbers, and expression levels of lncRNAs and mRNAs were observed to verify whether the predicted lncRNAs were consistent with the following general characteristics. Our data revealed that it was most common for lncRNAs to contain two exons, whereas mRNAs typically had more exons (Fig. 1b). The average length of lncRNAs was generally shorter than that of mRNAs (Fig. 1c). Figure 1 shows a box plot representing the lncRNA and mRNA expression levels based on the TPM model. The global expression trend of lncRNAs was toward notably higher expression (compared with the mRNAs), whereas the average expression level of lncRNAs was lower than that of mRNAs

### Analysis of lncRNA and mRNA expression levels in murine liver and spleen

**Fig. 2a–d:**
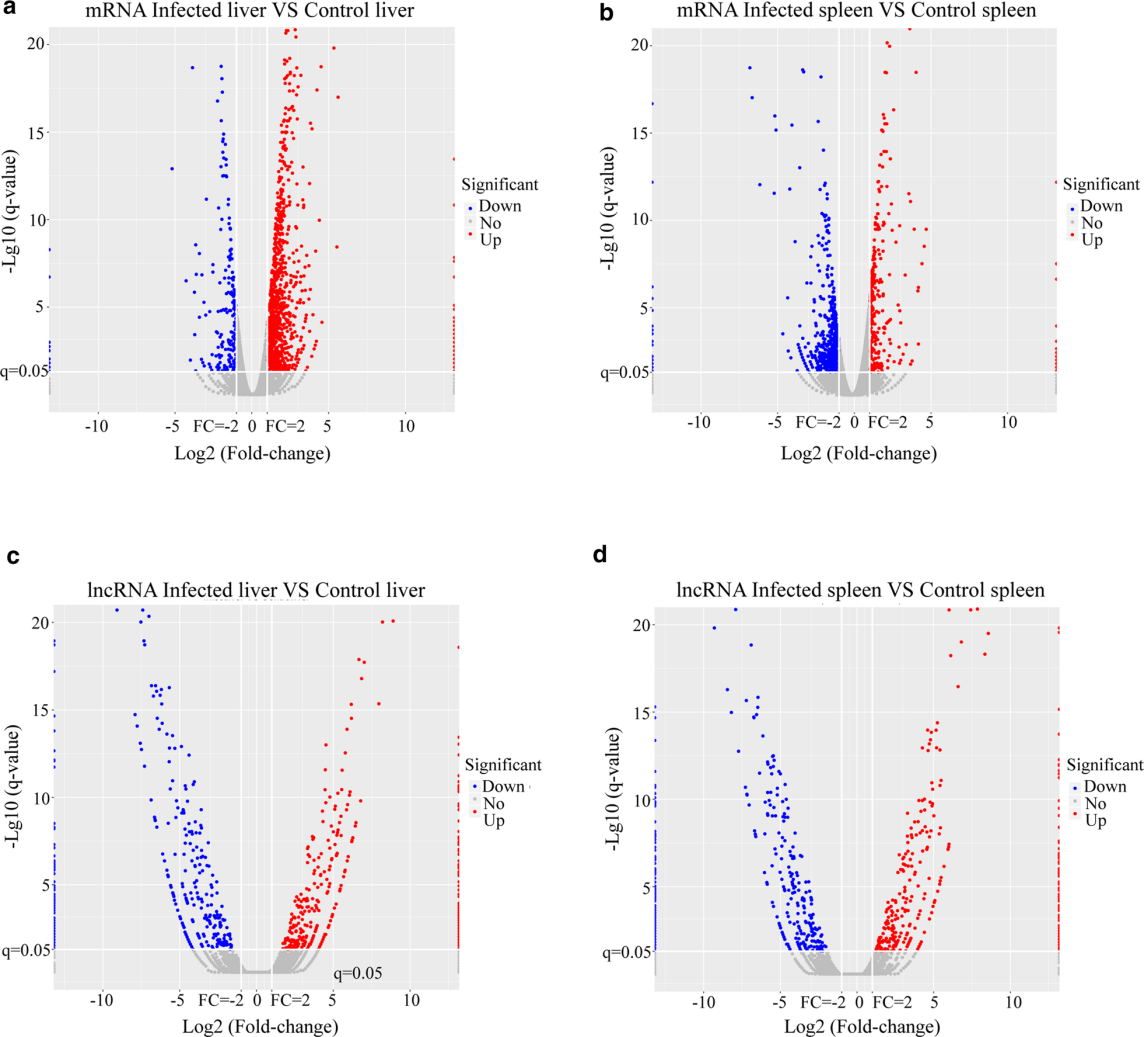
Differentially expressed mRNAs and lncRNAs in infected murine livers and spleens. **a** Volcano plot of differentially expressed mRNAs in livers of infected versus control mice. **b** Volcano plot of differentially expressed mRNAs in spleens of infected versus control mice. **c** Volcano plot of differentially expressed lncRNAs in livers of infected versus control mice. **d** Volcano plot of differentially expressed lncRNAs in spleens of infected versus control mice. The* x*-axis represents the log2 (fold change) values of the differentially expressed mRNAs/lncRNAs, and the* y*-axis represents the −log10 (*q*-value) values of the differentially expressed mRNAs/lncRNAs

To examine the potential biological functions of lncRNAs in *S. japonicum*-infected mice, we determined the lncRNA and mRNA expression profiles in the liver and spleen of control and *S. japonicum*-infected mice. We observed that 29,845 lncRNAs were expressed in the liver (Additional file 5: Table S5) and 33,788 lncRNAs were expressed in the spleen (Additional file 6: Table S6). In total, 19,065 mRNAs were expressed in the liver (Additional file 7: Table S7) and 21,634 mRNAs in the spleen (Additional file 8: Table S8). A pairwise comparison between the liver and spleen samples of control and infected mice revealed some mRNAs and lncRNAs that were differentially expressed. Up to 759 lncRNAs were differentially expressed in the *S. japonicum*-infected liver samples (compared with the control group), among which 378 were upregulated and 381 were downregulated. A total of 1,353 mRNAs were differentially expressed in the liver samples from *S. japonicum*-infected mice compared to the control liver samples. Among these mRNAs, 1126 were upregulated whereas 227 were downregulated. In the spleen, 789 differentially expressed lncRNAs (404 upregulated and 385 downregulated) were identified in the *S. japonicum*-infected spleen samples, compared with the control group. In addition, 1134 mRNAs were differentially expressed (354 upregulated and 780 downregulated) in the spleen samples of *S. japonicum*-infected mice, when compared to normal spleen samples. Significant differential expression was defined as probes with *q*-value ≤ 0.05 and fold change in expression of ≥ 2. TPM values were calculated to normalize the expression pattern of each mRNA and lncRNA, and upregulated and downregulated mRNAs and lncRNAs were defined based on a fold change in expression of ≥ 2 and a* q*-value ≤ 0.05, as depicted in the volcano plot shown in Fig. 2. Comparison of differentially expressed lncRNAs in the liver and spleen revealed liver-specific upregulation of 321 lncRNAs and liver-specific downregulation of 306 lncRNAs. Similarly, spleen-specific upregulation of 343 lncRNAs and spleen-specific downregulation of 314 lncRNAs was observed. Moreover, 132 lncRNAs were dysregulated in both tissue types. Among them, 43 lncRNAs were downregulated in both the liver and spleen, and 29 lncRNAs were commonly upregulated. In addition, 28 lncRNAs up-regulated in the liver were downregulated in the spleen, and 32 lncRNAs downregulated in the liver were upregulated in the spleen. Together, our results suggest that widespread differential regulation of lncRNAs occurs following *S. japonicum* infection in the liver and spleen of mice.

### Prediction of targets for differentially expressed lncRNAs

**Fig. 3:**
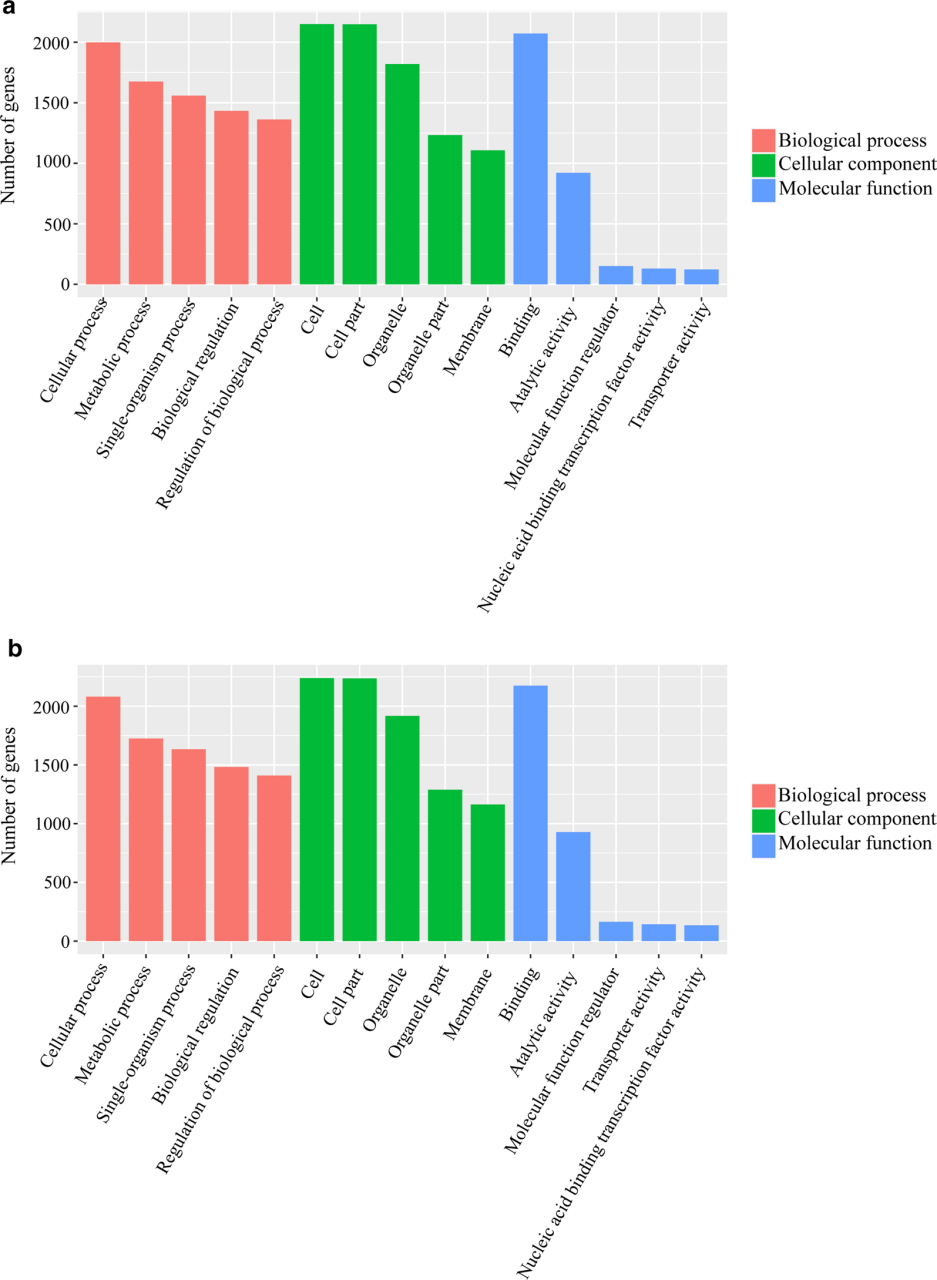
Enrichment of Gene Ontology terms for dysregulated lncRNA target genes in infected liver (**a**) and spleen (**b**) versus the corresponding control**.** The* horizontal axis* represents the annotation terms of interest, and the* vertical axis* shows the number of genes annotated to each term

**Fig. 4a, b:**
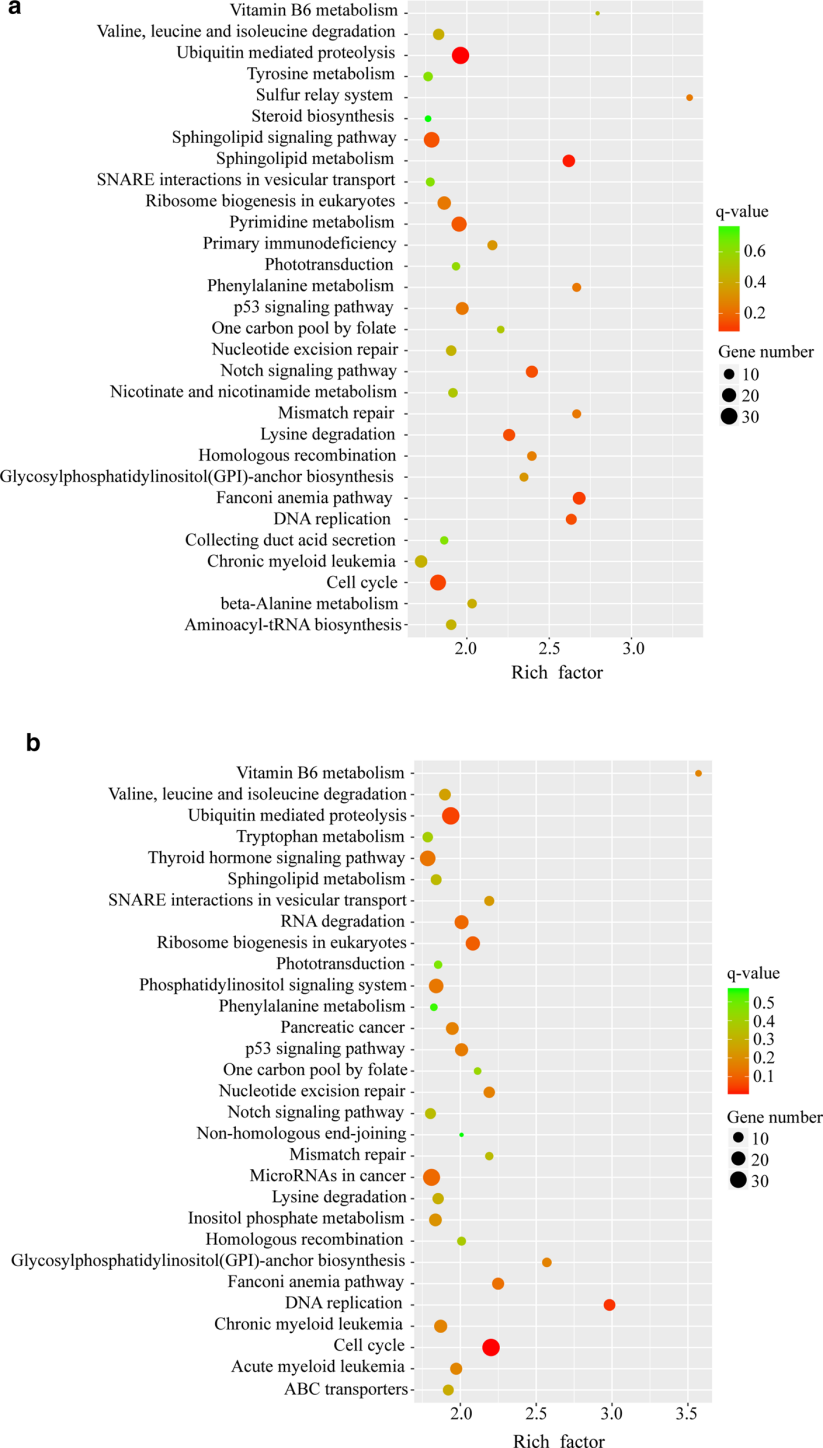
Kyoto Encyclopedia of Genes and Genomes (KEGG) pathway analysis of differentially expressed lncRNA targets in livers and spleens of infected mice. The top 30 KEGG terms associated with the differentially expressed lncRNA targets in liver (**a**) and spleen (**b**) of *S. japonicum*-infected mice versus control mice. The* vertical axis* shows the pathway names, and the* horizontal axis* shows the rich factors of KEGG pathway enrichment. The rich factor was calculated as (the number of differentially expressed genes associated with a specific pathway term/the number of all differentially expressed genes in the pathway database)/(the number of genes associated with a specific pathway term/the total number of genes in the pathway database). The* size* of each* dot* indicates the number of differentially expressed genes in the pathway, and the* color* corresponds to the different* q*-value range

To understand the possible functions of lncRNAs, we predicted the potential* cis* and* trans* targets of the differentially expressed lncRNAs. In *Schistosoma-*infected livers, we observed that 657 dysregulated lncRNAs had predicted targets (of which 247 were* trans*-regulated target genes), 553 lncRNAs had* cis*-regulated target genes, and 47 lncRNAs had both* trans*- and* cis*-regulated target genes. We found that 2548 mRNAs may be targeted by the differentially expressed lncRNAs and that, among these mRNAs, 454, 2157, and 41 were* cis*-regulated,* trans*-regulated, and both* cis*- and* trans*-regulated, respectively (Additional file 9: Table S9). Similarly, analysis of infected mouse spleens revealed 660 dysregulated lncRNAs, among which, 533 dysregulated lncRNAs had* cis*-regulated target genes, 267 had* trans*-regulated target genes, and 63 had* trans*- and* cis*-regulated target genes. Notably, 2763 mRNAs were predicted to be regulated by the dysregulated lncRNAs. Among these, 468 mRNAs were* cis*-regulated, 2268 mRNAs were* trans*-regulated, and 54 mRNAs were both* cis*- and* trans*-regulated (Additional file 10: Table S10). To further explore the functions of the lncRNAs during *S. japonicum* infection, differentially expressed lncRNA targets were selected for GO enrichment and KEGG pathway analysis. These dysregulated lncRNA targets were classified into three categories. The biological processes comprised 25 sub-categories, including cellular process and metabolic process; the cellular components comprised 20 sub-categories, including cell and cell part; and the most enriched GO terms in molecular functions were associated with binding and catalytic activity in both the liver and spleen (Fig. 3a, b; Additional file 11: Table S11; Additional file 12: Table S12). Furthermore, based on our KEGG pathway analysis, the differentially expressed lncRNA targets related to *S. japonicum* infection in the liver and spleen mainly participate in ubiquitin-mediated proteolysis (KEGG pathway mmu04120), cell cycle (KEGG pathway mmu04110), and metabolic pathways (KEGG pathway mmu01100). The top 30 enrichment pathways are shown in Fig. 4; Additional file 13: Table S13; and Additional file 14: Table S14.

### Prediction of lncRNA–mRNA networks

**Fig. 5a, b:**
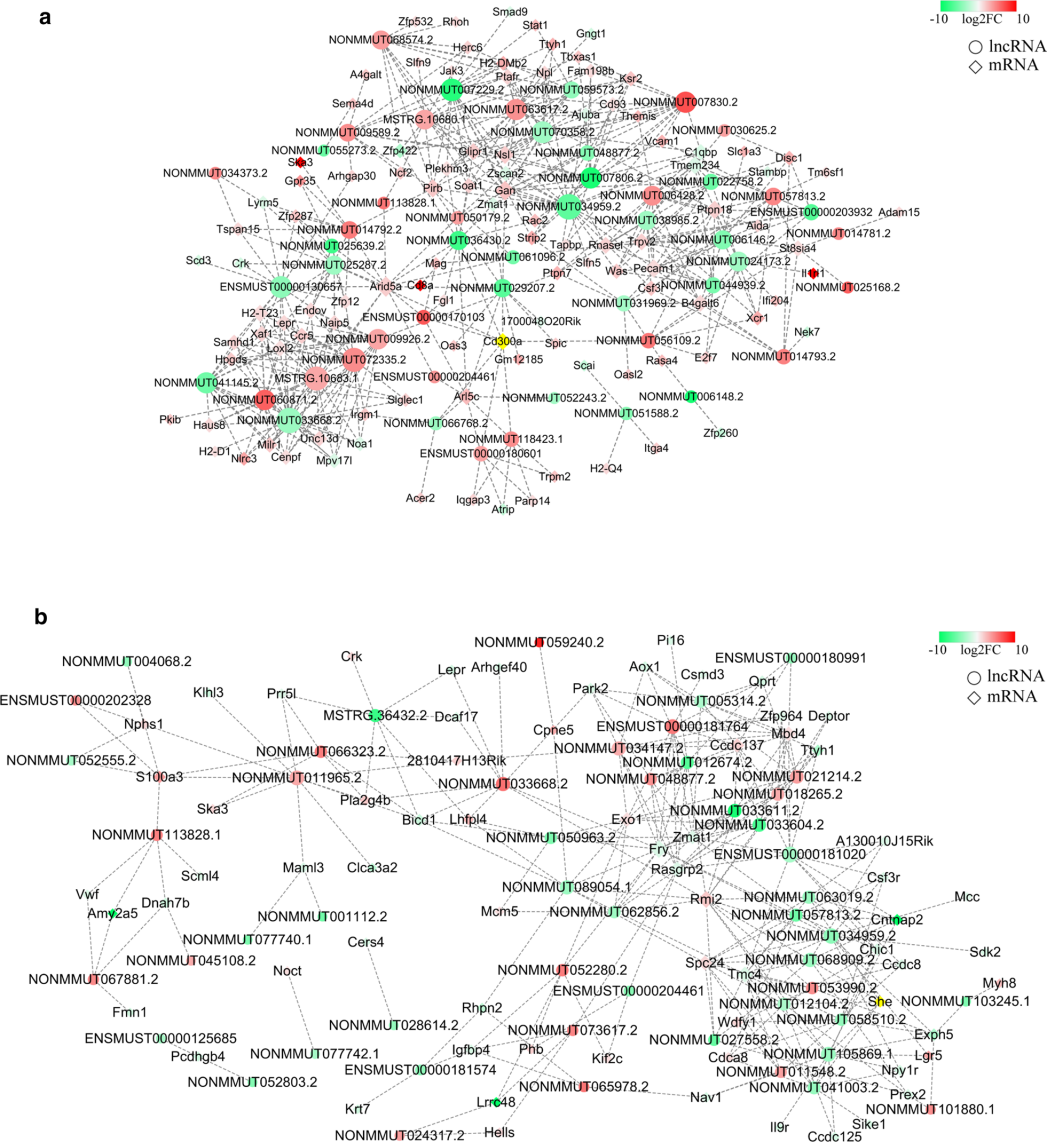
Co-expression network of differentially expressed lncRNAs and correlated mRNAs. **a** The 50 most differentially expressed lncRNAs and 107 dysregulated, correlated mRNAs in the livers of mice infected with *S. japonicum*. **b** The 50 most significantly expressed lncRNAs and 68 dysregulated, correlated mRNAs in the spleens of mice infected with *S. japonicum*. The* circles* represent lncRNAs and the* diamonds* represent target mRNAs. The* sliding color scale* from* green* to* red* indicates low to high expression, respectively. The* sizes* of the* circles* and* diamonds* indicate the number of associated genes

A co-expression network was constructed for the liver, which consisted of the top 50 differentially expressed lncRNAs and their correlated dysregulated mRNAs with 437 connection edges (Fig. 5a). Among these co-expressed lncRNA–mRNA pairs, 205 pairs were positively correlated, and 232 pairs were negatively correlated. In addition, we also built a network of lncRNA–mRNA pairs for the spleen, based on the top 50 differentially expressed lncRNAs and relative mRNAs, with 266 connection edges (Fig. 5b). Among them, 140 pairs were positively correlated, and 126 pairs were negatively correlated. The results revealed that lncRNAs may play significant regulatory roles in the pathogenesis of mouse liver and spleen infected by *S. japonicum.*

### RT-qPCR validation

**Fig. 6a–d:**
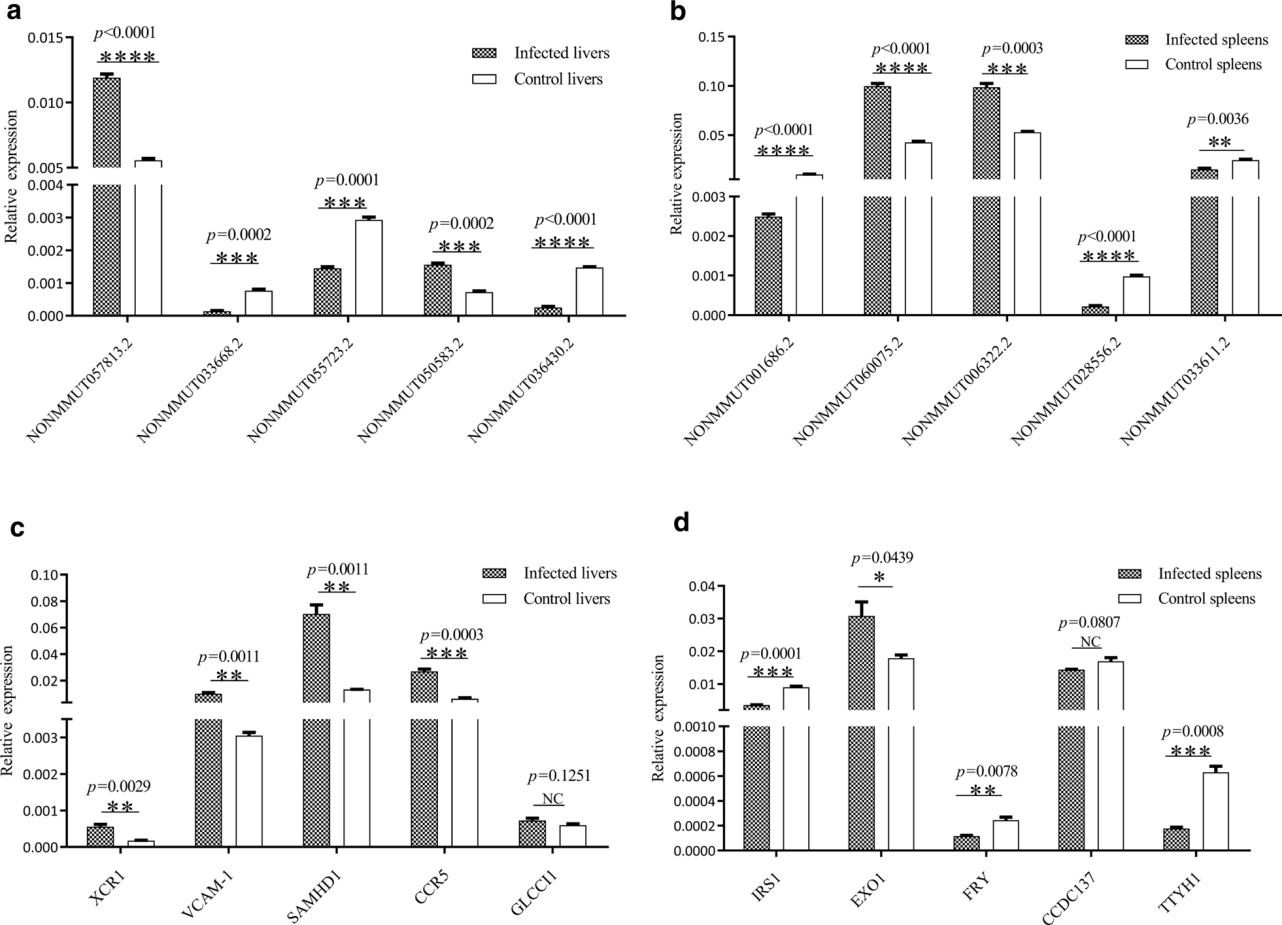
Verification of differentially expressed lncRNAs and their putative targets in livers and spleens of mice infected with *S. japonicum* by reverse transcription-quantitative real-time polymerase chain reaction (RT-qPCR). Relative expression levels of lncRNAs in livers (**a**) and spleens (**b**) of mice at 25 days post-infection. Relative expression levels of the putative targets for selected lncRNAs in livers (**c**) and spleens (**d**) of mice at 25 days post-infection. The relative expression levels were normalized to glyceraldehyde-3-phosphate dehydrogenase (*GAPDH*) gene expression.* XCR1* Chemokine C motif receptor 1,* VCAM1* vascular cell adhesion molecule 1,* SAMHD1* SAM domain and HD domain 1,* CCR5* chemokine C–C motif receptor 5,* GLCC1* glucocorticoid-induced transcript 1,* IRS1* insulin receptor substrate 1,* EXO1* exonuclease 1,* FRY* furry homolog* Drosophila*,* CCDC137*, coiled coil domain containing 137,* TTYH1* tweety homolog 1 *Drosophila*. The results are expressed as the mean ± SEM. * *p* < 0.05, ** *p* < 0.01 *** *p* <   0.01, *** *p* < 0.001, **** *p* < 0.0001,* NC* no statistically significant change

**Fig. 7a, b:**
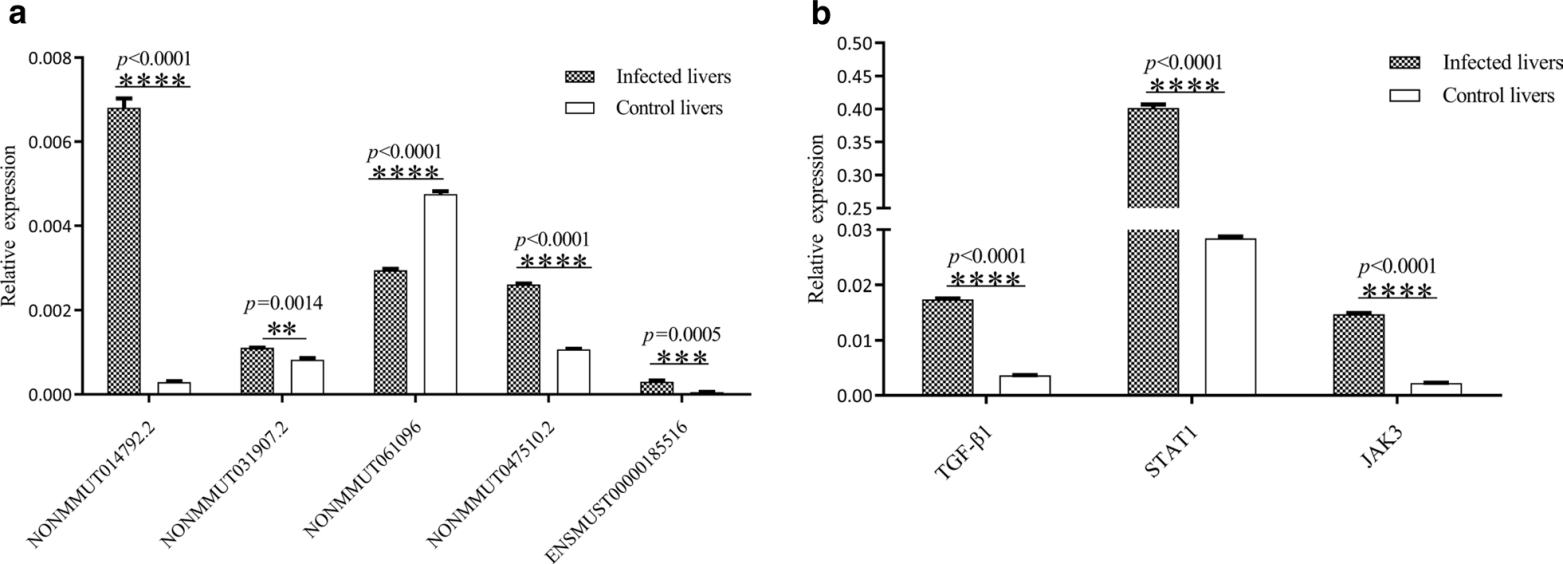
Differentially expressed lncRNAs and related putative targets in livers of mice infected with *S. japonicum* examined by RT-qPCR. **a** Relative expression levels of selected lncRNAs in the livers of mice infected with *S. japonicum* at 64 days post-infection. **b** Relative expression levels of several putative targets for lncRNAs in the livers of mice infected with *S. japonicum* at 64 days post-infection. The relative expression levels were normalized to *GAPDH* gene expression. The results are expressed as the mean ± SEM. **p < 0.01 ***p < 0.001, ****p < 0.0001. For abbreviations, see Figs. 1 and 6

To verify the reliability of the RNA-Seq results, we selected ten differentially expressed lncRNAs in the liver and spleen for RT-qPCR analyses. The sequences of selected lncRNAs were retrieved from the noncode database (www.noncode.org) and the primers were designed. The RT-qPCR results showed that the lncRNAs NONMMUT057813.2 and NONMMUT050583.2 were upregulated, whereas NONMMUT033668.2, NONMMUT055723.2, and NONMMUT036430.2 were downregulated in liver after *S. japonicum* infection (Fig. 6a, b). In addition, NONMMUT060075.2 and NONMMUT063222.2 were upregulated, whereas NONMMUT001686.2, NONMMUT028556.2, and NONMMUT033611.2 were downregulated in the spleen after *S. japonicum* infection, which was consistent with the RNA-Seq data and provided reliable validation of the sequencing data. We next selected several putative targets of LncRNAs to determine their expression in the livers and spleens of mice infected with *S. japonicum*. As shown in Fig. 6, apart from glucocorticoid induced transcript 1 (GLCC1; a putative target of NONMMUT055723.2), other selected targets such as chemokine C motif receptor 1 (XCR1; a putative target of NONMMUT057813.2), vascular cell adhesion molecule 1 (VCAM1; a putative target of NONMMUT057813.2), SAM domain and HD domain 1 (SAMHD1; a putative target of NONMMUT033668.2) and chemokine C C motif receptor 5 (CCR5; a putative target of NONMMUT033668.2) were significantly increased in the livers of mice infected with *S. japonicum*, while insulin receptor substrate 1 (IRS1; a putative target of NONMMUT001686.2), furry homolog *Drosophila* (FRY; a putative target of NONMMUT028556.2 or NONMMUT033611.2) and tweety homolog 1 *Drosophila* (TTYH1; a putative target of NONMMUT028556.2 or NONMMUT033611.2) were significantly reduced in the spleen of *S. japonicum-*infected mice. The results of the RT-qPCR confirmed the trend observed in the RNA-Seq data. In addition, we also evaluated the expression of several selected lncRNAs that may be involved in the regulation of TGFβ-1, JAK3, and STAT1, which are related to liver pathogenesis in *S. japonicum*-infected mice. As shown in Fig. 7, the levels of selected lncRNAs such as NONMMUT014792.2, NONMMUT031907.2, NONMMUT061096.2, NONMMUT047510.2 and ENSMUST00000185516 were aberrant in the livers of mice infected with *S. japonicum*. This altered expression was associated with increased expression of TGFβ-1, JAK3 and STAT1 (Fig. 7b).

## Discussion

lncRNAs play important roles in regulating gene expression at the transcriptional and post-transcriptional levels. Abnormal lncRNA expression is often involved in the pathogenesis and progression of many diseases [43]. In this study, we systematically analyzed genome-wide expression patterns of lncRNAs and mRNAs in liver and spleen samples from *S. japonicum*-infected mice at 25 days post-infection. We identified 38,313 lncRNAs, including 212 newly predicted lncRNAs. Among them, 759 and 789 lncRNAs were differentially expressed in the control and *S. japonicum*-infected mouse liver and spleen, respectively. Previous work has shown that lncRNA expression shows tissue-specific patterns [17]. Notably, we observed a higher number of lncRNAs differentially expressed in the spleen; however, the number of differentially expressed genes was higher in the liver, which suggests that there may be a large transcriptional difference between the spleen and the liver during *S. japonicum* infection. Ten dysregulated lncRNAs were identified and their putative targets selected randomly and verified via RT-qPCR; the qPCR results were similar to the RNA-Seq data.

Genome-wide analysis of different eukaryotic genomes has revealed thousands of extensively transcribed lncRNAs and short noncoding RNAs [43]. *S. japonicum* infection can cause damage to the host (such as liver fibrosis and hepatosplenomegaly) and can induce corresponding immune responses [4]. lncRNAs were recently reported to serve as functional regulators of fibrosis in different organs and tissues such as the liver, myocardium, kidneys, lungs, and peritoneum [44]. It is difficult to predict the functions of lncRNAs according to their nucleotide sequences owing to little knowledge of the relationships between the sequences or secondary structures of lncRNAs and their functions. However, we can assess the putative function of lncRNAs by analyzing the mRNAs that they regulate. In this study, we predicted the* cis* and* trans* mRNA targets of dysregulated lncRNAs. In total, 657 differentially expressed lncRNAs were predicted to target 2,548 protein-coding genes in the liver, and 660 differentially expressed lncRNAs were predicted to target 2,673 genes in the spleen. These findings indicated that lncRNAs might be related to *S. japonicum* infection and contribute to its pathogenesis, and might even be involved in parasite survival or virulence pathways. For example, the dysregulated lncRNAs including NONMMUT014792.2, NONMMUT031907.2, NONMMUT061096.2, NONMMUT047510.2 and ENSMUST00000185516 may be co-expressed with TGFβ-1, JAK3, and STAT1, which are reported to be important regulators of liver fibrosis [45]. Therefore, we hypothesized that these lncRNAs may play important roles in pathogenesis during *S. japonicum* infection. Moreover, we constructed molecular networks, based on the correlated targets of the dysregulated lncRNAs. Our results revealed that different lncRNAs can regulate the same gene and that the same lncRNAs can regulate different genes, suggesting that lncRNAs do not act alone, but rather work in groups with other lncRNAs and mRNAs.

GO enrichment analysis was employed to further examine the potential functions of differentially expressed lncRNAs. A previous study that employed GO analysis to identify fibrosis-associated genes targeted by miRNAs in *Mus musculus* showed that they were mainly related to protein-protein or protein-DNA binding [46]. Similarly, in this study, we found that target genes were also significantly enriched in terms of binding and protein binding. This finding indicated that *S. japonicum* infection may lead to the increased expression of associated genes and improved binding function. We noticed that chemoattractants, including those encoded by the target genes vascular endothelial growth factor A (VEGFA) and S100 calcium-binding protein A7A (S100A7A), comprised additional sub-categories in terms of the molecular functions operating in the spleen, suggesting the importance of chemoattractants in the mouse spleen during *S. japonicum* infection. Moreover, the expression levels of cell-adhesion molecules, including vascular cell adhesion molecule 1 (VCAM1) and platelet endothelial cell adhesion molecule 1 (PECAM1), were upregulated in the liver, but unchanged in the spleen. The differential expression of these genes and their correlated lncRNAs likely contribute to the pathogenesis of the mouse liver and spleen during *S. japonicum* infection.

A previous study focused on the KEGG pathway to identify miRNA targets during the late phase of *S. japonicum* infection, wherein it was discovered that they were mainly involved in pathways related to cancer, cytokine–cytokine receptor interactions, and mitogen-activated protein kinase signaling [47]; these results are similar to ours. In addition, our pathway analysis indicated that metabolic pathways, ubiquitin-mediated proteolysis, the phosphatidylinositide 3-kinase–protein kinase B (PI3K–Akt) signaling pathway, and other signaling pathways were also involved. Previous research showed that ubiquitin C plays a critical role in maintaining the tegument integrity of *S. japonicum* [48]; this also supports our findings. It is worth noting that a previous study demonstrated that the PI3K–Akt-signaling pathway is associated with parasite invasion [49], and we speculate that the PI3K–Akt-signaling pathway may also play a key role in *S. japonicum* infection. Moreover, many target genes associated with cell cycle progression were also identified as candidate lncRNA targets, indicating that cellular proliferation occurs in both the kidney and the liver during *S. japonicum* infection.

The expression profiles of lncRNAs are cell type, tissue, development stage, and disease specific, which may indicate their potential as diagnostic biomarkers for different diseases. Many lncRNAs have been correlated with diseases in mice, such as cancer and neurological diseases. Since lncRNAs can positively or negatively regulate gene transcription, they can potentially serve as therapeutic targets. There is growing commercial interest in the potential of lncRNAs as drug targets. In this study, we also found that hundreds of predicted lncRNAs were induced in infected liver and spleen samples, indicating that lncRNAs might contribute to the pathogenesis of *Schistosoma* infection. However, the functions of these lncRNAs remain unknown. Loss- and gain-of-function studies are needed to explore the biological significance of the expression of the lncRNAs that were dysregulated during *S. japonicum* infection in this study. Understanding the roles of lncRNAs in *Schistosoma* infection and pathogenesis might help in the development of novel diagnostic and therapeutic strategies.

## Conclusions

In summary, we analyzed global lncRNA expression levels in liver and spleen tissues of mice infected with *S. japonicum* by using RNA-Seq, and obtained numerous newly predicted and differentially expressed lncRNAs. Identifying differentially expressed lncRNAs and predicting their target mRNAs revealed the expression of genes associated with immunology and liver pathology, which may provide new insight into the pathogenic mechanisms of the host liver and spleen during *S. japonicum* infection. Further studies on the lncRNAs identified in this study will expand our understanding of genomic regulatory networks in the liver and spleen, which may provide new potential therapeutic targets for the treatment of *S. japonicum* infection.

## Supplementary information

**Additional file 1: Table S1.** Novel long noncoding RNAs (lncRNA) predicted in murine liver and spleen.

**Additional file 2: Table S2.** mRNA expression in murine liver and spleen.

**Additional file 3: Table S3.** lncRNA expression in murine liver and spleen.

**Additional file 4: Table S4.** lncRNA classification for murine liver and spleen.

**Additional file 5: Table S5.** lncRNA expression level in infected and control murine liver.

**Additional file 6: Table S6.** lncRNA expression level in infected and control murine spleen.

**Additional file 7: Table S7.** mRNA expression level in infected and control murine liver.

**Additional file 8: Table S8.** mRNA expression level in infected and control murine spleen.

**Additional file 9: Table S9.** Dysregulated lncRNA target genes in infected and control murine liver.

**Additional file 10: Table S10.** Dysregulated lncRNA target genes in infected and control murine spleen.

**Additional file 11: Table S11**. Gene Ontology (GO) annotation of target genes in infected and control murine liver.

**Additional file 12: Table S12.** GO annotation of target genes in infected and control murine spleen.

**Additional file 13: Table S13.** Kyoto Encyclopedia of Genes and Genomes (KEGG) enrichment of target genes in infected and control murine liver.

**Additional file 14: Table S14.** KEGG enrichment of target genes in murine spleen.

## Data Availability

All data supporting the findings of this article are included in the main article and its additional files. The raw sequencing data have been deposited in the NCBI under project no. PRJNA629486.

## Abbreviations

lncRNAs: Long noncoding RNAs
RT-qPCR: Reverse transcription-quantitative real-time polymerase chain reaction
miRNA: MicroRNA
TGFβ-1: Transforming growth factor beta 1
JAK3: Janus kinase 3
STAT1: Signal transducer and activator of transcription 1
PI3K–Akt: Phosphatidylinositide 3-kinase-protein kinase B
GO: Gene Ontology
KEGG: Kyoto Encyclopedia of Genes and Genomes
CPC: Coding Potential Calculator
CNCI: Coding-Non-Coding Index
TPM: Transcripts per million
GAPDH: Glyceraldehyde-3-phosphate dehydrogenase
